# Treatment options for patients with HR+/HER2− advanced breast cancer during the COVID-19 pandemic: dose reduction of ribociclib does not diminish efficacy

**DOI:** 10.1007/s10549-020-05674-7

**Published:** 2020-05-20

**Authors:** Joyce O’Shaughnessy

**Affiliations:** 1 US Oncology Research, Inc., The Woodlands, TX USA; 2 Texas Oncology/Baylor University Medical Center, Dallas, TX USA

**To the editor,**

The COVID-19 pandemic poses unprecedented challenges to the field of oncology. I read with great interest the recent recommendations outlined by Deitz et al. for patients with breast cancer during the COVID-19 pandemic [1]. For patients with hormone receptor-positive/human epidermal growth factor receptor 2-negative (HR+/HER2−) metastatic breast cancer, it is recommended that use of targeted oral therapies be weighed against the risk of adverse events and that dose reductions can minimize treatment-related toxicities. This was followed by the statement “dose reduction of palbociclib does not diminish efficacy.”

Currently, there are three CDK4/6 inhibitors approved for the treatment of HR+/HER2− metastatic breast cancer. To provide additional information for health care professionals in their decision-making, I would like to point out that dose reduction of the CDK4/6 inhibitor ribociclib also does not diminish efficacy. An analysis of patients with advanced breast cancer in the phase III MONALEESA-2, -3, and -7 trials who received ribociclib as initial endocrine-based therapy for advanced breast cancer showed that median progression-free survival was comparable between patients who had no dose reduction vs patients with ≥ 1 dose reduction (MONALEESA-2: 27.7 months vs 25.3 months; MONALEESA-3: not estimable vs not estimable; MONALEESA-7: 23.8 months vs 27.5 months) [2]. It has also been reported separately that ribociclib demonstrated a significant overall survival benefit over endocrine therapy alone in phase III trials (MONALEESA-3 and -7; MONALEESA-2 overall survival data is immature at this time) [3]. In all three trials, the most frequent adverse event (all grades) for patients with no dose reduction and ≥ 1 dose reduction was neutropenia. The most common reason for dose reduction was an adverse event.

Awareness of all available options may help in making treatment decisions for individual patients. I hope the information provided here can aid in that process.
